# Réflexions sur la tribune « Mayotte, une île enfin exempte de paludisme? » de J.-F Lepère, L. Collet, *et al.*

**DOI:** 10.48327/mtsi.v3i1.2023.341

**Published:** 2023-03-15

**Authors:** Sixte Blanchy

**Affiliations:** 19 rue Auguste Lançon, 75013 Paris, France

La tribune publiée en février 2023 « Mayotte, une île enfin exempte de paludisme? »^1^1LEPÈRE J-F, COLLET L, IDAROUSSI A-B, P RADINES B. Mayotte, Une île Enfin Exempte De Paludisme? Med Trop Sante Int. 2023. Fév. 15 3 (1). 3(1):mtsi.v3i1.2023.289. doi: 10.48327/mtsi.v3i1.2023.289 a le mérite d'actualiser les données publiées ces 20 dernières années sur la morbidité palustre dans le 101^e^ département français. Cependant, elle ne me semble pas répondre totalement à la question posée dans le titre sur les aspects épidémiologiques et entomologiques et ne tient pas suffisamment compte de la situation géographique de Mayotte dans l'océan Indien à proximité de zones toujours impaludées (Fig. 1).

**Figure 1:**
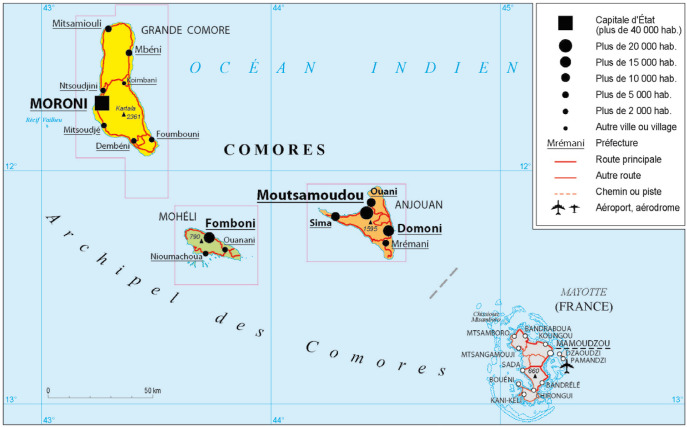
Les quatre îles de l'archipel des Comores ©Archives du Ministère de l'Europe et des Affaires étrangères – La Courneuve, 2018 The four islands of the Comoros archipelago ©Archives of the Ministry for Europe and Foreign Affairs (France) – La Courneuve, 2018

La situation actuelle est la résultante de l'histoire épidémiologique du paludisme aux Comores (à Mayotte et dans les trois autres îles sœurs, notamment à Anjouan) et du développement socio-économique important de Mayotte ces 20 dernières années.

Le développement a entraîné des modifications écologiques pour les vecteurs, une urbanisation et une modification de l'habitat, une forte augmentation du niveau de vie, une amélioration de l'accès aux soins. Tous ces éléments sont défavorables à la prolifération des vecteurs et favorisent une prise en charge rapide et de qualité des accès palustres de la majorité de la population.

On trouvera une bibliographie plus complète sur le plan historique dans les références ciaprès.

Les risques d'une recrudescence ou même d’épidémies de paludisme me semblent sous-estimés:

Aucune donnée entomologique sur l’écologie, la répartition entre espèces, la densité des vecteurs et leur sensibilité aux insecticides, la disparition d'anciens gîtes (du fait de la pollution par exemple), mais aussi la création de nouveaux (quid des gîtes proches des estuaires des rivières, abreuvoirs naturels?), n'est apportée dans cette tribune ou dans sa bibliographie alors que le climat et les gîtes majeurs des anophèles vecteurs ont probablement peu varié;

La forte immigration en provenance des trois autres îles des Comores et également de plus en plus de Madagascar (notamment par Nosy Bé) ainsi que d'Afrique continentale, importe chaque jour des gamétocytes sans aucun contrôle réalisé (ni probablement possible), d'autant plus que l'accès au diagnostic et aux soins des clandestins, aux conditions de vie précaires, représentant une part très importante de la population, est limité même en cas d'accès palustre par la chasse qui leur est faite;

À noter les fréquents voyages des résidents de Mayotte dans les autres îles et à Madagascar avec un risque d'infestation d'autant plus important que la « mémoire » du paludisme s'estompe;

La perte de la prémunition du fait de la faible transmission actuelle rend la population particulièrement sensible, enfants et adultes, à des infections palustres entraînant une morbidité et également un risque épidémique comme cela a déjà été le cas historiquement, par exemple après un passage de cyclone multipliant les gîtes larvaires des anophèles.

Ces risques réels entraînent la nécessité d'une surveillance continue et probablement renforcée aux plans épidémiologique, parasitologique et entomologique, et pas seulement de la morbidité.

Les principales mesures pourraient être:

- Maintenir l'accès aux soins des clandestins (diagnostic et traitement sans risque d'arrestation sur la route, dans les dispensaires et à l'hôpital);
- Le développement de la surveillance entomologique compte tenu des modifications rapides des possibilités et de la productivité de gîtes larvaires à proximité des maisons, « bangas » et campements dans les champs, de l'utilisation des insecticides agricoles et domestiques;
- Le suivi des résistances aux anti-malariques et aux insecticides;
- La coopération pour le développement avec les autres îles de l'archipel.

## Petite Bibliographie Historique

- Blanchy S, Benthein F. Chimiosensibilité *in vivo* de *Plasmodium falciparum* en République Fédérale Islamique des Comores. Bull Soc Pathol Exot Filiales. 1989;82(4):493-502. https://gallica.bnf.fr/ark:/12148/bpt6k97755652/f53.
- Blanchy S, Julvez J, Mouchet J. Stratification épidémiologique du paludisme dans l'archipel des Comores. Bull Soc Pathol Exot. 1999;92(3):177-84. https://pathexo.societe-mtsi.fr/documents/articles-bull/BullSocPatholExot-1999-92-3-177-184.pdf.
- Feillet N, Agnamey P, Brasseur P, Druilhe P. Résistance *in vivo* à la chloroquine de *Plasmodium falciparum* à Anjouan (Comores). Bull Soc Pathol Exot. 1993;86(1):48-51. https://gallica.bnf.fr/ark:/12148/bpt6k97755652/f53.
- Julvez J, Blanchy S. Le paludisme dans les îles de l'archipel des Comores. Éléments historiques et géophysiques, considérations épidémiologiques. Bull Soc Pathol Exot Filiales. 1988;81:847-853. https://gallica.bnf.fr/ark:/12148/bpt6k9775375r/f857.
- Le Bras J, Simon F, Ramanamirija JA, Calmel MB, Hatin I, Deloron P, Porte J, Marchais H, Clausse JL, Biaud JM, Sarrouy J, Guiguemde TR, Carme B, Charmot G, Coulaud JP, Coulanges P. Sensibilité de *Plasmodium falciparum* aux quinoléines et stratégies thérapeutiques: comparaison de la situation en Afrique et à Madagascar entre 1983 et 1986. Bull Soc Pathol Exot Filiales. 1987;80(3 Pt 2):477-89. https://gallica.bnf.fr/ark:/12148/bpt6k97755793/f483.
- Petrarca V, Sabatinelli G, Di Deco MA, Papakay M. The *Anopheles gambiae* complex in the Federal Islamic Republic of Comoros (Indian Ocean): some cytogenetic and biometric data. Parassitologia. 1990 Dec;32(3):371-80. www.researchgate.net/publication/21024484_The_Anopheles_gambiae_complex_in_the_Federal_Islamic_Republic_of_Comoros_Indian_Ocean_some_cytogenetic_and_biometric_data.

## Liens D'intérêts

L'auteur ne déclare aucun lien d'intérêt.

